# Challenges in the Management of Narcolepsy in a Resource Limited Setting: A Case Report

**DOI:** 10.7759/cureus.58143

**Published:** 2024-04-12

**Authors:** Joseph Yaria, Tobi Olusakin

**Affiliations:** 1 Medicine, University College Hospital Ibadan, Ibadan, NGA; 2 General Practice, Vine Branch Medical Center, Ibadan, NGA

**Keywords:** cataplexy, nigeria, narcolepsy misdiagnosis, narcolepsy type 1, narcolepsy

## Abstract

The management of Narcolepsy, from the initial presentation to the long-term management and follow-up, remains a challenging endeavor, especially in developing climes. Worldwide, it has been recognized as a medical condition that is frequently associated with initial misdiagnoses, and delays in definitive management, further highlighted, in resource-limited settings like Nigeria where issues are further compounded by social, cultural, and political factors. In this report, we aim to shed some light on the peculiar challenges encountered by clinicians in Nigeria, and in other similar settings, in the process of diagnosis and management of narcolepsy.

We present a case of a 17-year-old male teenager with Narcolepsy Type 1 (NT1) who had been previously managed as a case of Juvenile Absence Epilepsy in various centers prior to presentation at our facility. The symptoms began two years prior to presentation at our outpatient clinic, and they were excessive daytime sleepiness, cataplexy, and sleep paralysis. The symptoms were corroborated by laboratory parameters - reduced mean sleep latency (conducted in an improvised sleep laboratory), and a low cerebrospinal fluid (CSF) hypocretin level. The patient was initially placed on Modafinil for excessive daytime sleepiness and a trial of Fluoxetine for the Cataplexy. However, due to the scarcity of Modafinil, behavioral modifications - scheduled sleep naps and sleep hygiene - were eventually employed.

Narcolepsy is a debilitating illness, and consequently, the far-reaching effects of these challenges must be understood. It is important that concerted efforts be made towards improving the overall quality of care received by patients from the early identification to the treatment of narcolepsy in the Nigerian healthcare system.

## Introduction

Narcolepsy is a neurological disorder that has been understood to be caused by the loss of, or dysfunction of orexin neurons in the hypothalamus [1]. It is an uncommon and underdiagnosed disorder that has a strong association with Human Leucocyte Antigen (HLA) DQB1*0602. Other predisposing factors include autoimmunity and triggers such as infections and brain trauma. A link between emotional stress and Narcolepsy has also been suggested by some authors. Narcolepsy is often characterized by excessive daytime sleepiness, hallucinations, sleep paralysis, cataplexy, and sleep fragmentation. Recent advances categorize the disease as Narcolepsy type 1 (NT1) or Narcolepsy type 2 (NT2) narcolepsy, depending on the presence of cataplexy or the lack thereof. Due to the sleep disruption that occurs, the disease often affects the overall body functioning, thereby leading to problems in the professional, social, and emotional aspects of the patient’s life [2].

In Nigeria, a hospital prevalence rate of 0.026 has previously been reported, with males being slightly more affected than females [3]. Despite the debilitating nature of the condition, a delay in its diagnosis exists, especially in Africa [4]. This occurs as a result of a mélange of factors including, but not limited to, the poor health-seeking behavior that is rampant in the populace, the low index of suspicion for the condition, and the dearth of qualified medical professionals and appropriate diagnostic facilities. Even in cases where diagnoses have been made, management remains a difficult task. This case report aims to describe and shed some light on the delay and difficulties experienced in the diagnosis and management of narcolepsy in Nigeria.

## Case presentation

The patient was a 17-year-old male teenager who was referred to the Neurology outpatient service of our hospital. He complained of recurrent falls, and what was described as “brief alterations in consciousness.” He had begun to notice these symptoms two years earlier after his university roommate witnessed him having a brief episode of loss of consciousness lasting less than a minute. The episodes were often associated with head drops, occasional squat drops, and/or drop attacks - falling like a pack of cards. The patient, however, denied losing consciousness, insisting that he was just too weak to respond in those situations. He admitted having a feeling of heaviness, and/or having a tingling sensation in his head prior to the falls. The patient did not experience loss of vision, tonic-clonic movements, or bladder/bowel incontinence during those episodes. There was also no prior history, or family history, of epilepsy or cardiac conditions. Prior to presentation, he had been managed in various hospitals as a case of juvenile absence epilepsy and had been placed on a few anti-seizure drugs prior to presentation at our center, but symptoms persisted.

At subsequent clinic visits, further details obtained clarified the events even more, thus revealing a relation of the episodes to emotional situations and states such as laughter, excitement, and pleasant surprises. Additionally, the patient admitted having excessive daytime sleepiness despite getting seven to eight hours of night-time sleep. His mother also corroborated that he could not be left alone without dozing off and sleeping for hours. She had attributed this to boredom, as he only came home during the holidays. He admitted falling asleep during monotonous activities like lectures and talks. He, however, could stay awake if he was active or engaged in activities that he really enjoyed. The patient also recalled that he had started having sleep paralysis and weight gain three years prior to presentation, but had no complaints of snoring, intermittent awakenings during sleep, hallucinations, hypersexuality, hyperphagia, hyperorality, obsessive-compulsive symptoms, skin-picking behavior, impulsivity, or symptoms suggestive of rapid eye movement (REM) sleep behavioral disorder.

There was no history of sustained weakness in any specific part of the body, double vision, imbalance, facial sensory deficits, dysphagia, voice changes, skin lesions, cough, fever, breathlessness, or nausea. Throughout the patient’s history, there were no symptoms suggestive of depression, hypothyroidism, or substance use disorder. There was also no family history of any sleep disorders.

The patient had a background history of degenerative myopia, anisometropia, and left exotropia, all of which began when he was five years old and was managed by an ophthalmologist with corrective lenses. He also suffered from a mild speech impediment - stammering, and a limb length discrepancy. Physical examination revealed no abnormalities, and on mental status examination, he appeared to be indifferent about his condition, though he admitted to having some distress due to his symptoms.

**Figure 1:**
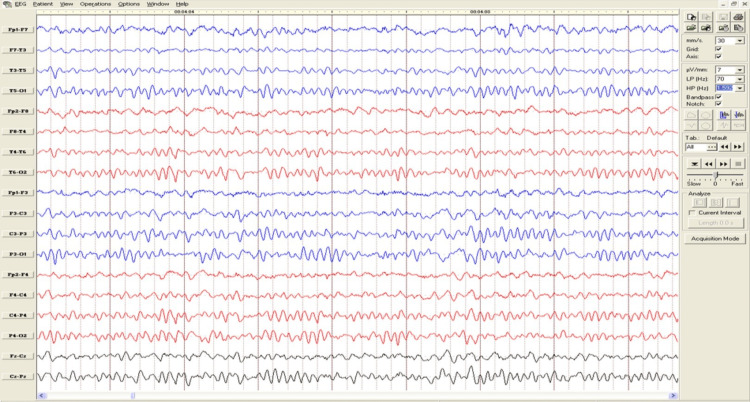
A normal EEG reading obtained during the initial assessment of the patient. EEG: Electroencephalogram.

**Figure 2:**
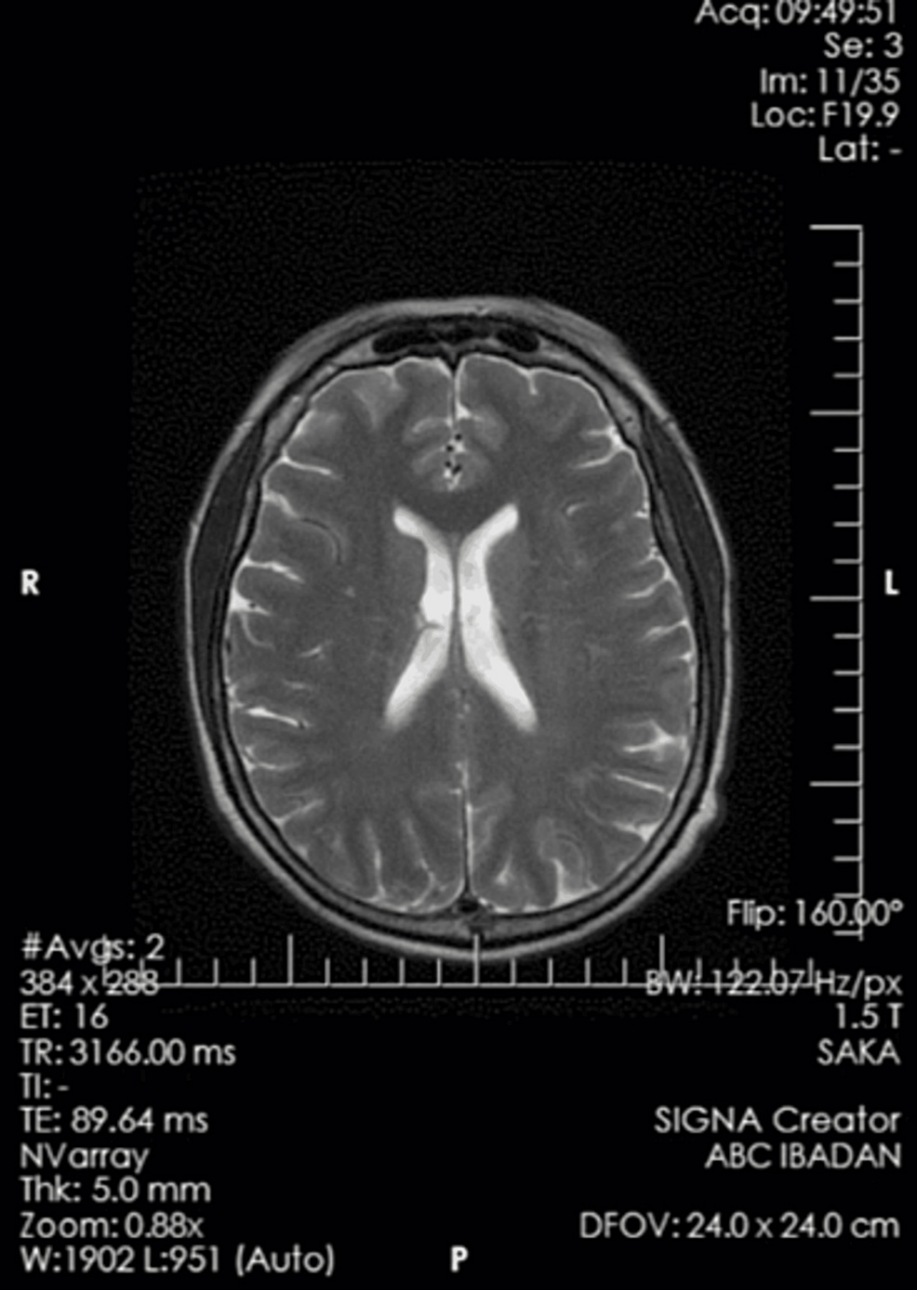
An axial view of a T2WI brain MRI scan with normal findings. T2WI: T2-weighted images; MRI: Magnetic resonance imaging.

Routine investigations including complete blood count, random blood glucose, malaria parasite screen, and serum electrolytes - were normal. He had an electroencephalogram (EEG) done, as shown in Figure 1, which showed no epileptiform or non-epileptiform abnormalities. A magnetic resonance imaging (MRI) scan was eventually requested (Figure 2), which also revealed no abnormalities.

After further details were obtained at subsequent clinic visits, the patient was admitted for an adapted multiple sleep latency test and/or event monitoring. The patient was asked to ensure at least six hours of sleep per night for at least three days prior to the presentation. Due to the lack of a standard sleep laboratory, a routine EEG machine was used for recording in a dark and quiet room with comfortable temperature levels (Video 1). An EEG montage (Fz-Cz, C4-M1, Oz-Cz) was created with electrodes placed for an electro-oculograph. Sleep latency was defined as the time from lights out to sleep onset. Sleep onset was defined as the first 30-second epoch containing more than 15 seconds of stage one sleep changes - when more than 50% of the alpha waves are replaced with low-amplitude fast activity, reduced muscle artifacts, or vertex waves. After four sleep trials, a mean sleep latency of 6mins 39s was obtained with individual sleep latencies of 2mins 2s, 1min 45s, 20mins, and 2mins 49s. Sequel to the results, a cerebrospinal hypocretin quantification was requested; samples were shipped out of the country, and the result came out weeks later as <30 pg/mL.

**Video 1:** A video of the patient undergoing an improvised sleep study

A diagnosis of Narcolepsy with cataplexy was then made using the International Classification of Sleep Disorders, Third Edition (ICSD-3), diagnostic criteria for NT1.

After the diagnosis, the patient and his parents were counseled on the condition, its symptoms, and the treatment options. The behavioral adjustments recommended included regular sleeping time of at least eight hours, and avoidance of long or unscheduled naps during the day. He was, instead, allowed to have two naps per day, each lasting a duration of about 20 minutes; this was found to improve his functional quality of life. Due to the scarcity of medications like Pitolisant and Solriamfetol, the patient was placed on Modafinil 200mg daily (before noon) for excessive daytime sleepiness, and a trial of Fluoxetine 20mg for the cataplexy. After the commencement of the regimen, a reduction in the intensity and frequency of symptoms was observed; even though he was able to continue on Fluoxetine, the use of Modafinil was not sustained as it was notoriously difficult to source for. Methylphenidate and stimulants such as Buproprion were also mostly unavailable at this time. As such, the excessive daytime sleepiness was eventually managed with behavioral modification alone - scheduled sleep naps and sleep hygiene.

## Discussion

According to the Diagnostic and Statistical Manual of Mental Disorders, Fifth Edition (DSM-5) and the International Classification of Sleep Disorders, Third Edition (ICSD-3), a diagnosis of narcolepsy requires that symptoms of excessive daytime sleepiness occur at least three times per week for at least three weeks, and at least one of the following criteria: cataplexy, a low cerebrospinal fluid (CSF) hypocretin level, and a sleep study that demonstrates abnormal REM sleep latency [5]; the criteria were met by the patient. Classically, narcolepsy manifests as a tetrad of excessive daytime sleepiness, cataplexy, hypnagogic hallucinations, and sleep paralysis. Three of these symptoms were present in the case. Non-cardinal symptoms like sleep paralysis and weight gain [2], amongst others, were also observed in the case.

The ability to recognize the symptoms of narcolepsy oftentimes eludes medical professionals. Rosenberg and Kim report that, according to the AWAKEN survey, less than 10% of primary care providers and 22% of sleep specialists correctly identify all five narcolepsy symptoms [6]. This informs the consideration of using validated measures to identify patients with features of narcolepsy. Scales such as the Epworth Sleepiness Scale (both the child and adolescent version) [7], Cataplexy Questionnaire [8], Ullanlinna Narcolepsy Scale [9], and the five-item Swiss Narcolepsy Scale [10] should be considered, especially in low-resource settings like Nigeria.

While it is understandable that narcolepsy is rare, it is estimated that about 50% of individuals are undiagnosed [1]. The delay in the diagnosis of narcolepsy has been well documented, with most participants receiving a diagnosis more than one year after symptom onset [11]. A median diagnostic duration of 10.5 years has been reported by Morrish [12], and 8.9 years by Taddei [13]. Interestingly, a duration as long as 61 years has also been reported [12]. It is, therefore, unsurprising that an old study reported the median onset of symptoms as 16 years, but the median age of diagnosis as 33 years [14]. Higher CSF orexin levels, the absence of cataplexy, and a prolonged duration of symptoms, that is, pediatric onset of symptoms, have been shown to independently predict a delay in diagnosis [11-13]. Other variables linked to a delay in diagnosis include longer intervals between daytime sleepiness and cataplexy onset, lower cataplexy frequency, shorter durations of irresistible daytime sleep, lower daytime REM sleep propensity, and being female [15]. It should be noted that patient-related factors, especially delay in presentation, contribute to the delay in diagnosis. Taddei et al. reported a mean time gap between disease onset and first medical consultation of 3.2 years [13].

Misdiagnosis of narcolepsy is common; a review of the Nexus Narcolepsy Registry reported a misdiagnosis rate of close to 60% [14]. The initial diagnosis is usually made by a general practitioner, while other diagnoses come from neurologists, sleep clinics, or respiratory physicians [16]. Common conditions with symptoms that overlap with narcolepsy include seizures, hypotension, psychiatric disorders, schizophrenia, night terrors, depression, insomnia, obstructive sleep apnea, and idiopathic hypersomnia [17]. Interestingly, some other conditions that have been mistaken for narcolepsy include iron deficiency, burnout, psychosomatic disorders, and poor sleep hygiene [13]. Narcolepsy is often misdiagnosed as epilepsy in many clinical environments, more so in places that are significantly deficient in medical resources. This is reflected in the case presented where the patient was initially managed as a case of Juvenile Absence Epilepsy. A lack of clear-cut symptoms, or situations where inadequate clinical details are extracted - as seen in the earlier clinic visits in our facility - further make it difficult to clinch a correct diagnosis. The patient’s description of the cataplectic events could be easily misconstrued as syncopal or seizure episodes. It is also important to note that, in a country with a doctor-to-patient ratio of 1:5,000, asking for a detailed consultation in a single visit, in a crowded outpatient clinic, is often considered a herculean task.

The lack of a functioning standard sleep laboratory in the state added to the difficulty in making a diagnosis. This inspired the use of a regular electroencephalogram as an adaptable option for a modified multiple sleep latency test. These challenges were, also, not helped by the underdeveloped sphere of sleep medicine in the region, and in Africa as a whole [18]. Cerebrospinal levels of hypocretin could also not be assayed in the country, and samples had to be flown overseas - a situation not unique to Nigeria. These issues put a lot of pressure on out-of-pocket health financing for the patients thereby limiting access to laboratory evaluation. In a significant proportion of the clinical settings in Nigeria, the lack of qualified personnel and appropriate laboratory resources ensures that it remains difficult to make a definitive diagnosis via mean sleep latency test and CSF analysis for hypocretin, therefore, greatly contributing to the burden of misdiagnosis and delays in management. Simple options like actigraphy and sleep diary may be adapted to help objectively determine hypersomnia.

Owolabi and Ogunniyi have reported a case of narcolepsy has been reported from Nigeria in the past [3], and the hospital prevalence rate has been reported as 0.026. Management has been via non-pharmacological approaches - scheduled sleep naps, and pharmacological ones - Methylphenidate [3]. Decades later, these medications still remain largely unavailable and have not been replaced by newer options. Out of all the central nervous system (CNS) stimulants recommended, Modafinil was the only option available, but it was also not sustainable. The use of caffeine seems to be the only readily available option, but it is not a practical one. For the management of cataplexy, a selective serotonin reuptake inhibitor (SSRI) was chosen; insomnia, a common side-effect of this medication, may be beneficial in this scenario. This appeared to have worked, as the patient admitted to having an improved quality of life, despite occasional cataplexy episodes still occurring.

Other newer classes of wake-promoting compounds have been developed in recent years, with others expected to surface in the near future. Amongst them is Pitolisant, a new wake-promoting agent which is a selective histamine H3 receptor inverse agonist [4]. It acts pre-synaptically, activating histamine neurons in the process [19]. Solriamfetol (JZP-110), a new high-potency wake-promoting agent, selectively inhibits dopamine and norepinephrine without promoting the release of monoamines [20]. To ensure the availability of these newer and more effective options, there is a need to integrate sleep disorders across the country, or in regions, to improve treatment access, specialization, and early intervention.

## Conclusions

Narcolepsy is a medical condition of importance that significantly impacts the quality of life of affected individuals. Its presence, unfortunately, oftentimes goes unnoticed in resource-poor settings such as Nigeria where the available medical resources ensure that efforts at early identification, diagnosis, and treatment are frustrated. Despite its rare occurrence, the disease occurs in the Nigerian setting and has debilitating effects on survivors. Thus, there is a need for concerted effort and support from both national and international entities to facilitate the early identification, diagnosis, and treatment of narcolepsy and other sleep disorders in Nigeria and other similar resource-poor settings.
